# Water Resources Utilization and Protection in the Coal Mining Area of Northern China

**DOI:** 10.1038/s41598-018-38148-4

**Published:** 2019-02-04

**Authors:** Shuning Dong, Bin Xu, Shangxian Yin, Yong Han, Xiaodong Zhang, Zhenxue Dai

**Affiliations:** 1 Xi’an Research Institute of China Coal Technology & Engineering Group Corp, Xi’an, 70054 China; 2 College of Geoscience and Surveying Engineering, China University of Mining & Technology (Beijing), Beijing, 100083 China; 3 Hebei State Key Laboratory of Mine Disaster Prevention, North China Institute of Science and Technology, Beijing, Yanjiao 101601 China; 4 School of Environmental Science and Engineering, Shandong University, Qingdao, 266237 China; 5 College of Construction Engineering, Jilin University, Changchun, 130026 China; 6 Earth and Environmental Sciences Division, Los Alamos National Laboratory, New Mexico, 87545 USA; 7 Key Laboratory of prevention technology of Water Hazard in Coal Mines, Xi’an, 70054 China

## Abstract

This study investigates multiple types of water resources in the western mining area in China, their supply-demand balance by using the same dimension gray recurrence dynamic model GM (1, 1), and water content coefficients of mines to ease water supply-demand contradiction. A multi-objective programming model is proposed for optimal water resources allocation management. Optimal technical schemes for water resources allocation among different users are obtained. The optimization model improves upon the previous studies by using water demand and water supply forecast. Coordinated development for mining safety, mine water utilization, and remediation and control of water environment is achieved.

## Introduction

China’s water resources are relatively scarce, in general, and water resources per capita are only a quarter of the world’s average^1–4^. Water resource problems are more prominent in the arid and semi-arid areas in northern China^5–8^, where most of coal mines of China are located^9–11^. Moreover, coal mining causes serious groundwater losses, which exacerbates the contradiction between water shortages and socio-economic development^12–17^. The biggest difference between water resource utilization and management in coal mining area and other region is that the supply of water resources for coal mining area is severely affected by coal mining and the mine draining, which could change water circulation system by the following aspects: changing the transformation relation between surface water and groundwater, accelerating the infiltration rate of rainfall and surface water, reducing evaporation and complicating water circulation^18^. Water resources protection and management are urgently needed for dealing with the contradiction between water supply and demand in coal mines in northern China^19–22^.

Protection of water resources in the processes of coal mining includes mainly three methods for achieving effective utilization of mine water: water conservation mining, underground reservoir storing, and simultaneous exploitation of coal and groundwater as resources. These methods have been widely applied for mine water utilization in coal mines in northern China^23–26^, including water conservation mining in Shennan Mining Area, Shaanxi^27^, underground reservoir construction and mine water utilization in Daliuta coal mine, Shaanxi^28^, and simultaneous exploitation of coal and groundwater in Lu’an coalfield, Shanxi^29^. These different methods were developed based on different geological and hydrogeological conditions of the different coal mines.

The optimal management technique has been widely used in many fields, such as engineering management and decision-making^30,31^, and it has been applied for water resources management in coal mines in the past two decades, but, recently it shifted from solely relying on water supply to paying attention to increasing water supply, saving water resources, and managing water demand, as well as from single objective to multiple objective management^32–36^. Many places in China have conducted the related study with various techniques of water resources utilization to ease the contradiction between supply and demand and solve drought emergency problems^37–41^. The multi-objective decision management model has been initialized to model and optimize future water resources utilization^35,42,43^, since this method can build a model of regional multi-objective optimal allocation of water resources with considering the factors such as economy, society, and environment, which reflects the principles of fairness, priority, efficiency and comprehensive benefits. Also, it is useful for enhancing the economic and social benefit and ecological environment protection^44,45^. For examples, Wu (1995) derived a comprehensive theory of mine water utilization, named “balance of drainage and water-supply for environmental protection”, which was successfully applied in Jiaozuo coal mine in China^46^. Management of mine water resources in the mine areas currently mainly focuses on multi-objective decision-making, while direct forecast of water demand and mine water drainage (which was routinely used many years ago) are insufficient for multi-objective optimal management of water resources in coal mines^47,48^. This study uses the Dongsheng coal mining area as an example to derive the multi-objective model for water resources optimal management in coal mines in northern China.

The Dongsheng mining area, located near the contiguous zone in the north of Shaanxi Loess Plateau and southeast of Maowusu desert, is one of the world-class coal production bases in China. Continuous increasing of development scale of coal resources in this area has caused the changes of aquifer structure and conditions of groundwater recharge, flow and discharge, which in turn results in significantly declined water levels, dry aquifers, reduced or even disappeared flow of some springs^49^. These are due to decreased precipitation and increased mine drainage and water consumption of agriculture and industry every year. All of them lead to the contradiction among the water demand increase of the coal industry and other energy and chemical industries, the shortage of regional water resources, and protection of eco-environment in the mining areas^50^. Therefore, the study on water resources protection and optimal management is of vital importance for the sustainable use of water resources and balance environmental protection and industrial development in these regions.

In this study, a multi-objective optimization management model is developed to obtain optimal solutions for water resources utilization constrained by quantities of surface water, groundwater, and mine water in Dongsheng mining area. It is of great significance for rational development, comprehensive utilization and scientific management of water resources in the study area. The results are helpful for providing references for water resources protection and management in coal mine areas in the arid or semi-arid zones.

## Material and Methods

### Study area

**Figure 1:**
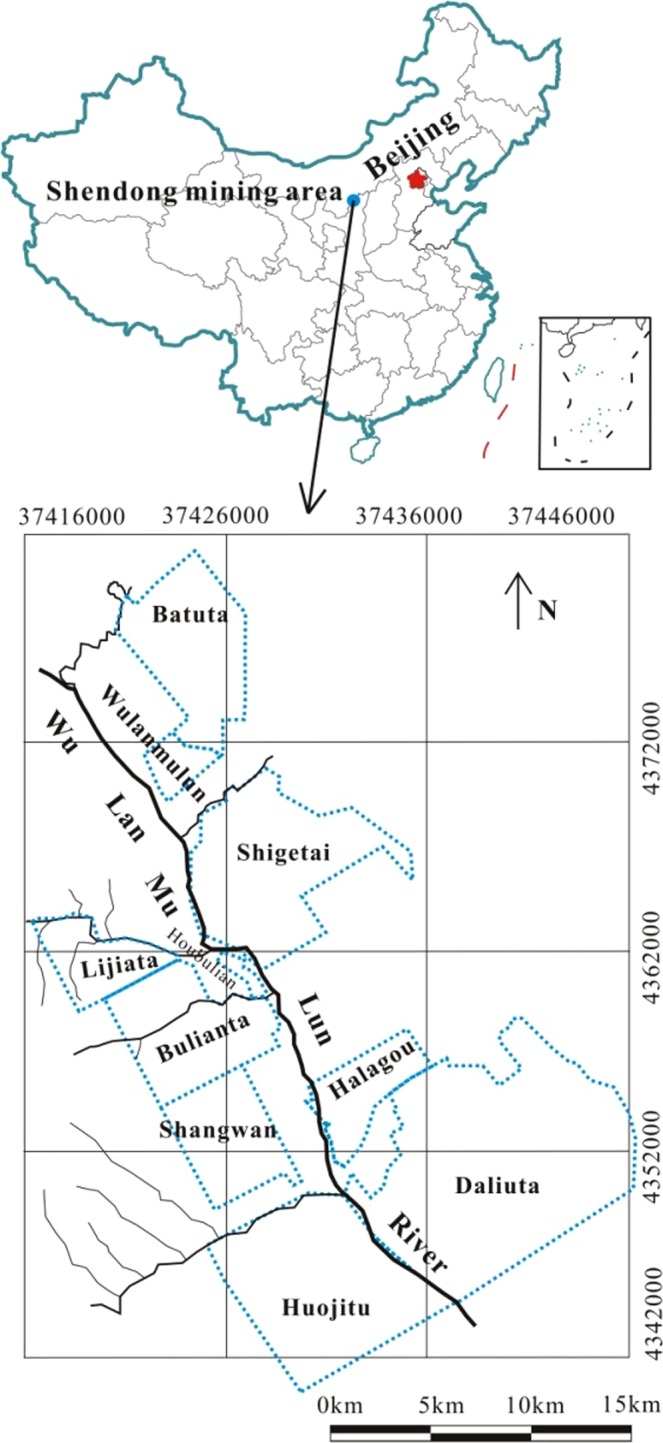
Location of the study area.

The study region, Dongsheng mining area, has an area of over 500 square kilometers (Fig. 1), where three kinds of water resources are available, including surface water, groundwater, and mine water. Surface water includes the Wulanmulun river, lakes, reservoirs, ponds, and some other surface water bodies. Mine water includes mine water drainage from mines Daliuta, Huojitu, Shangwan, Bulainta, Halagou, Shigetai, and Wulanmulun.

### Multi-objective optimization model

The multi-objective optimization model aims to systematically consider water sources allocation, including water quantity, water quality, and the characteristics of main water users, to achieve the highest water supply guarantee rate and the lowest cost of water supply. The decision variables are the supplied water amounts from various water sources to different water users in different sub-regions.

The study area is divided into eight sub-regions, including Daliuta, Bulianta, Shangwan, supply system of coal liquefaction, Halagou, Wulanmulun, Shigetai, and Huojitu. The water users include production, living, industry, and virescence. The water sources include tap water, industrial reuse water, and virescence recycled water. The number of decision variables in the study area is 96 (8 × 4 × 3). The multi-objective optimization model of water resources in the mining area has two objective functions.

Objective 1: to meet the maximum water-supply needs of each sub-region.

1 $${\rm{\max }}\,{f}_{1}(x)=\sum _{k=1}^{8}\sum _{j=1}^{4}\sum _{i=1}^{3}{x}_{ijk}$$

Objective 2: to minimize the total water supply cost in the study area.

2 $${\rm{\min }}\,{f}_{2}(x)=\sum _{k=1}^{8}\sum _{j=1}^{4}\sum _{i=1}^{3}{x}_{ijk}{\alpha }_{j}$$

where *f*_1_*(x)* is water supply amount in the study area; *f*_2_*(x)*is the total water supply cost in the study area; *x*_*ijk*_ is the amounts of water supply from water source *i* to user *j* in sub-region *k*; *α*_*j*_ is the cost coefficient of unit water supply from water source *j*; *i* is index for the number of water source types (*i* = 1, 2, 3); *j* is index for the number of water user types (*j* = 1, 2, 3, 4); and *k* is index for the number of the sub-region (*k* = 1, 2, …, 8).

Constraint conditions are the law, rules, and regulations the model relies on when seeking the solution, only under the definite constraint conditions, the calculation results are effective. The present model has 5 constraints:

- constraints of water demand
  The largest supply of water should not be greater than 120% of water demand on the basis of meeting the needs of each user, in order to save water and reduce water supply costs. Water demand constraints include water demand constraints of production, living, industry, and virescence as follows:
  3 $${Q}_{k-1}\le {x}_{11k}+{x}_{21k}\le 1.2\cdot {Q}_{k-1}$$
  4 $${Q}_{k-2}\le {x}_{12k}\le 1.2\cdot {Q}_{k-2}$$
  5 $${Q}_{k-4}\le {x}_{14k}+{x}_{24k}+{x}_{34k}\le 1.2\cdot {Q}_{k-4}$$
  where *Q*_k−1_ is water demand for production; *Q*_k−2_ is water demand for living; *Q*_k−4_ is water demand of virescence.
- constraints of water supply capacity of each sub-region
  6 $$\sum _{k=1}^{8}\sum _{j=1}^{4}{x}_{1jk}\le {Q}_{T-W}+{Q}_{I-W}$$
  7 $${x}_{21k}+{x}_{23k}+{x}_{24k}\le {Q}_{R-W}$$
  8 $${x}_{34k}\le {Q}_{V-W}$$
  where *Q*_T-W_ is water supply capacity of the tap water; *Q*_I-W_ is water supply capacity of each mine’s own independent water resource; *Q*_R-W_ is total water supply capacity of industrial reused water; *Q*_V-W_ is total water supply capacity of virescence recycled water.
- constraints of water quality
  Contaminants need to meet emission standards and cannot exceed the total emissions.
  9 $${C}_{kj}^{r}\le {C}_{0}^{r}$$
  10 $$\sum _{k=1}^{K}\sum _{j=1}^{J(k)}0.01{d}_{j}^{k}\,{p}_{j}^{k}\,(\sum _{i=1}^{I(k)}{x}_{ijk}+\sum _{c=1}^{I(k)}{x}_{cjk})\le {W}_{0}$$
  where ${C}_{kj}^{r}$ is the concentration of pollutant *r* from user *j* in sub-region k; ${C}_{0}^{r}$ is the standard concentration of pollutant *r* for pollutant discharge; *W*_0_ is total permitted pollution discharge capacity.
- non-negative constraints of decision variables
  11 $${x}_{ijk}\ge 0$$
  The decision variables involved in the optimation calculation, i.e., the water supply to different users in different subareas should be bigger or equal to zero.
- zero constraints of decision variables

12 $${x}_{31k}={x}_{32k}={x}_{33k}$$

13 $${x}_{22k}=0$$

Following the standard of modeling and the principle of water supply by quality, formula (12) expresses that the reused water for afforestation is not used in production, livelihood, and industry, its corresponding variable is zero and not involved in the optimization calculation formula (13) expresses that the industrial reused water is not used in livelihood, its corresponding variable is zero and not involved in the optimization calculation.

According to the objective of the current project, this paper has chosen the tool *fgoalattain* in Matlab to solve the multi-objective problem. *fgoalattain* solves the multiobjective goal attainment problem, which is one formulation for minimizing a multiobjective optimization problem^51^:

14 $$\begin{array}{ll}\mathop{{\rm{minmize}}}\limits_{x,\gamma } & {\rm{\gamma }}\,\,\,{\rm{such}}\,{\rm{that}}\,F(x)-w{\rm{eight}}\cdot {\rm{\gamma }}\le goal\\  & \,\,\,\,\,\,\,A\cdot x\le b\\  & \,\,\,\,\,\,\,Aeq\cdot x=beq\\  & \,\,\,\,\,\,\,c(x)\le 0\\  & \,\,\,\,\,\,\,ceq(x)=0\\  & \,\,\,\,\,\,\,lb\le x\le ub\end{array}$$

where *x*, *weight*, *goal*, *b*, *beq*, *lb*, and *ub* are vectors, *A* and *Aeq* are matrices, and *c(x)*, *ceq(x)*, and *F(x)* are functions that return vectors. *F(x)*, *c(x)*, and *ceq(x)* can be nonlinear functions.

The formulations for multiobjective optimization have been fully discussed in the Standard Algorithms chapter for showing how to use Matlab optimization toolbox^51–54^.

## Results

### Mine Water Discharge at National Level

According to the data of National Bureau of Statistics in China, during the 10^th^ to 12^th^ “Five-Year National Plan” periods (from 2005 to 2020), national wastewater discharge amounts have grown linearly by 10 billion m^3^ per five-year. However, the growth of coal mine wastewater discharge amount has declined gradually, compared with the previous five-year plan (from 2001 to 2004). And the growth rate of coal mine wastewater discharge from the10^th^ to 12^th^ “Five-Year” has decreased by 169.2%, 32%, and 17.3%, respectively. At the same periods, the rate of national coal mine water utilization has increased by 35.2%, 59.2%, and 75.5%, respectively.

### Water Resources Situation in the Study Region

There are rich coal resources but scarce water resources in northern China. With rapid social and economic development, regional conflicts between supply and demand of water resources have become increasingly prominent. The utilization of the available mine water includes simply mine water treatment and reuse for coal mining, which is far from the concept of coordinated development and optimal decision on environmental protection and utilization of mine water resources.

According to a field investigation conducted in October and December 2006^55^, the amount of surface water resources is about 1.197 × 10^8^ m^3^, including about 1.17 × 10^8^ m^3^ of mean annual runoff of the Wulanmulun River. There are 47 surface water bodies including lakes, reservoirs, and ponds (Table 1), with a total water surface area of 5.91 × 10^5^ m^2^ and a total surface water volume of 2.70 × 10^6^ m^3^, in which the Wulanmulun lake is the largest one with a water surface area of 4.0 × 10^5^ m^2^ and a volume of 2.2 × 10^6^ m^3^.

**Table 1:** The water surface areas and volumes of lakes, reservoirs, and ponds in the study region.

Name	Daliuta	Huojitu	Shangwan	Bulianta	Halagou	Shigetai	Wulanmulun	Sum
Water surface area (m^2^)	6820.5	12421.1	2716.2	57336.8	31500.0	80000.0	400000.0	590794.6
Water volume (m^3^)	14361.8	17804.2	7324.5	94464.5	82750.0	280000.0	2200000.0	2696705.0

The main recharges of groundwater in the study region are rainfall infiltration and lateral runoff, and the main discharges are spring discharge, coal mine water drainage and base outflow to the Wulanmulun river.

The balance equation in the study area during the balance duration (one year) is:

15 $${\rm{\Delta }}\varepsilon ={Q}_{rf}+{Q}_{lr}-{Q}_{spr}-{Q}_{wi}-{Q}_{bf}$$

where Δ*ε* is the change amount of groundwater reserve; *Q*_*rf*_ is the recharge amount from rainfall infiltration; *Q*_*lr*_ is the amount of underground lateral runoff; *Q*_*spr*_ is the amount of spring discharge; *Q*_*wi*_ is the amount of coal mine water drainage; and *Q*_*bf*_ is the amount of base outflow to the Wulanmulun river.

For the study of regional water balance, generally it is necessary to select a complete hydrological year as the balance period, and median water year is the best for the hydrological year^56^. Thus, 2004 (from 1975 to 2018) was selected to conduct the study of water balance with precipitation guarantee rate of 50%. It can be calculated from the above formulas that the total amounts of recharge, discharge, and the change in water storage volume are 80.8, 82.29, and minus 1.49 million m^3^ in this study area during the balance period, respectively (Table 2). The recharge amounts from the rainfall infiltration and the lateral runoff are 75.98 and 4.82 million m^3^, respectively. The spring drainage, the mine water drainage, and the base outflow to the Wulanmulun river are 33.27, 12.43, and 36.59 million m^3^, respectively (Table 2). Groundwater budget is negative balance in the first year of the balance period, with a reduced amount of groundwater storage of about 1.49 million m^3^.

**Table 2:** The groundwater balance calculation during the balance period.

Balance elements	Recharge amount		Balance element	Discharge amount	
	(million m^3^)	(%)		(million m^3^)	(%)
Rainfall infiltration	75.98	94.03	Spring discharge	33.27	40.40
Lateral runoff	4.82	5.97	Coal mine water drainage	12.43	15.10
/	/	/	Base outflow to Wulanmulun river	36.59	44.50
Total recharge	80.80	100	Total discharge	82.29	100

The mine water drainage of these seven coal mines is 1470.50 m^3^/h and the total annual amount is 12.43 million m^3^ (Table 3), which are increasing year by year. In addition, the total surface water area in these seven mines is 130.63 million m^2^ and the total volume is 39.31 million m^3^. Therefore, the mine water resources have great potential to be exploited and utilized as an alternative water source.

**Table 3:** The mine water resources of seven coal mines in the study region.

Mine name	Daliuta	Huojitu	Shangwan	Bulianta	Hala river	Shigetai	Wulanmulun	Sum
Drainage rate (m^3^/h)	338.3	136.8	87.8	504.3	80.8	155.0	167.5	1470.5
Total annual drainage (million m^3^)	2.97	1.09	0.76	4.42	0.71	1.26	1.47	12.43

According to the analysis above, the total volume of water resources of surface water and groundwater in the study region is 130.64 million m^3^, which was calculated by the sum of the amount of surface water resources (119.70 million m^3^) and groundwater resources (80.8 million m^3^) minus the amount of double-counting 69.86 million m^3^. The amount of double-counting contains spring discharge 33.27 million m^3^ and base outflow to Wulanmulun river 36.59 million m^3^ (Table 2).

### Water Demand Prediction

The tap water and industrial recycled water demands in the following 15 years are forecasted by a same dimension gray recurrence dynamic model GM (1, 1), where the supply amount of tap water and industrial water during 2000–2004 is used as original data to simulate and predict future water demands, and to check the accuracy of GM (1, 1) model. The simulation results of current water consumption listed in Table 4 shows that the probability of small error, *P* is bigger than 0.95, the variance ratio, *C* is smaller than 0.35, indicating that the fitting accuracy meets the standard of class one^57^.

**Table 4:** Simulation results of current water consumption.

Year	Original value (m^3^/d)	Fitting value (m^3^/d)	Residual error	Relative error (%)
2000	20,822	20,822	0	0
2001	21,260	20,817	443	2.09
2002	20,966	21,639	−673	−3.21
2003	22,761	23,500	−739	−3.25
2004	23,622	24,120	−498	−2.11
*P* = 1,*C* = 0.12				

As shown in Table 5, both *C* (<0.35) and *P* (>0.95) of each year of the prediction meet the standard of class one. According to the principle of posterior error test, when the development grey number, *a*, of GM (1, 1) is smaller than 0.3, the model can be used in medium and long term prediction. Parameter *a* listed in Table 5 is all smaller than 0.1, which means that GM (1, 1) model has some practical value, and can be used in long-term prediction.

**Table 5:** Prediction results of tap water demand during the planning period.

Year	Predicted value (m^3^/d)	Development grey number *a*	C	P
2005	30,600	−0.097	0.107	1
2008	33,205	−0.094	0.102	1
2010	35,800	−0.086	0.128	1
2015	46,457	−0.0788	0.186	1
2020	54,203	−0.0730	0.248	0.95

**Figure 2:**
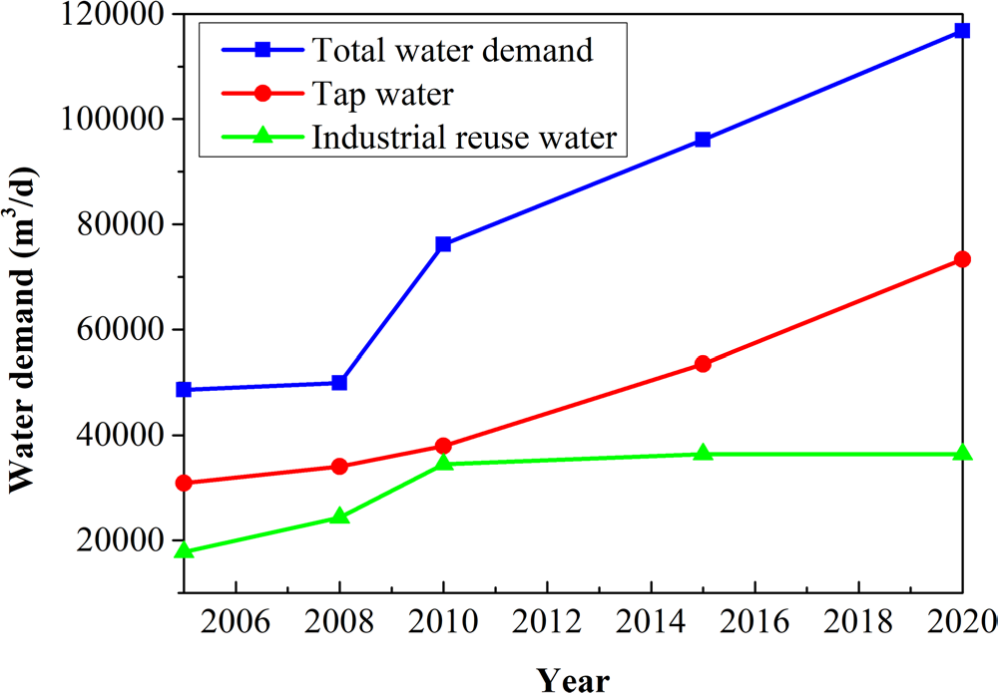
Predicted water demand in the balance period.

Demands of all kinds of water resources will increase from 4.86 × 10^4^ m^3^/d in the first year (2005) of the balance period to 11.68 × 10^4^ m^3^/d in 2020, in which tap water demand increases by 4.24 × 10^4^ m^3^/d, and industrial recycled water demand increases by 1.86 × 10^4^ m^3^/d (Fig. 2).

**Figure 3:**
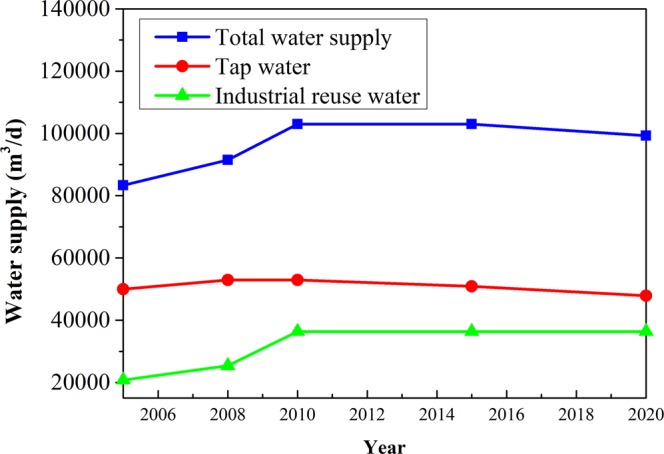
The forecasted water supply in the balance duration.

The water supply amount is predicted during the balance period, which increases from 8.33 × 10^4^ m^3^/d in 2005 to 1.03 × 10^5^ m^3^/d in 2010, then remains roughly constant at 1.03 × 10^5^ m^3^/d from 2010 to 2015, and finally decreases to 9.93 × 10^4^ m^3^/d in 2020 (Fig. 3). The predicted water supply yield rooted mainly in the *“Eleventh Five Years Plan”* and *the Prospect Objective to 2020 of Coal Industry of Shendong Branch of China Shenhua Coal Group Corporation”*. In terms of the proportion of water supply, the proportion of tap water supply will decrease from 71% in the first year to 56.8% in the fifteenth year, while the proportion of industrial recycled water supply will increase from 29.1% in the first year to 43.2% in the fifteenth year. Therefore, the results indicate that the utilization rate of mine water resource will increase in the future, which can not only alleviate the contradiction between supply and demand of water resources, but also save money and improve the enterprises’ economic and social benefit.

### Water Yield of Mine Prediction

**Figure 4:**
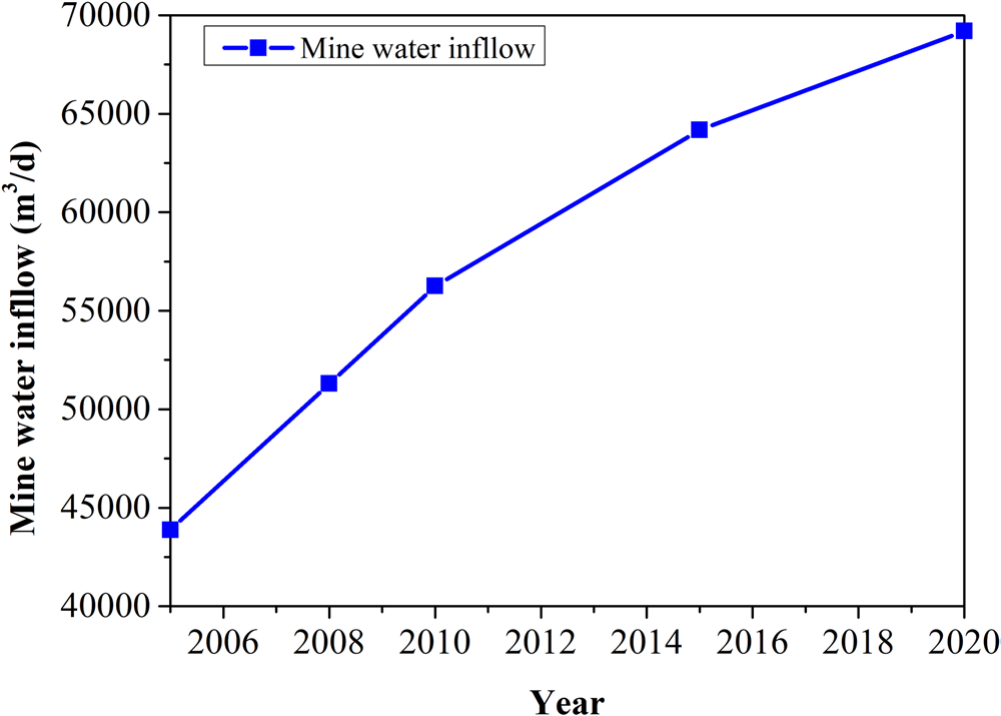
The prediction results of mine water drainage in the Shendong mining area.

The mine water was predicted by using the water content coefficient of mine, one of the hydrogeological analogy methods^58^:

16 $${K}_{p}=\frac{Q(t)}{P(t)}$$

where *K*_*p*_ is the water content coefficient of mine; *Q(t)* is the mine water drainage quantity, and *P(t)* is the coal yield in the same period (usually a year). The prediction results of mine water are shown in Fig. 4, which indicates that mine water drainage will increase yearly in each mine of the Shendong mining area. The total drainage amount will increase from 4.39 × 10^4^ m^3^/d in 2005 to 6.92 × 10^4^ m^3^/d in 2020.

### Optimal Allocation of Water Resources

In view of the water supply and demand conditions in Shendong mining area, a multi-objective decision analysis was conducted to deal with the contradiction of water use in each mine, and determine the optimal allocation of water resources.

The water supply in the study area is mainly composed of groundwater provided by the water source of each spring area, surface water; mine water and reuse water are provided by domestic sewage treatment plants. According to the current water use mode of the mining area and water quality, water resources are divided into tap water supply, industrial reuse water, and virescence recycled water. Among them, industrial reuse water is composed of mine water reuse water, mainly for industrial, production and other water sectors in the study area; virescence recycled water is composed of the treated domestic sewage from Daliuta and Heitagou domestic sewage treatment plants, Liuta and Huojitu underground water treatment plants for watering lawns and trees. The water quality was used to classify the water resources in this work.

In order to realize the full utilization of water resources and to achieve cost minimization on the basic of the water supply to meet the demand in the mining area, the priority sequence was carried out according to the users. In other words, the demand for production was given priority, followed by the demand for life, industry, and irrigation.

The water demand data of each user in the sub-district in 2008, 2010, 2015, 2020 and the water supply capacity data of each water source were brought into constraints of the previous section, and a constraint matrix was formed. Then, the constraint matrix was substituted in the multi-objective programming function fgoalattain of Matlab as a parameter. Finally, the results of multi-objective optimization of water resources of each user in 2008, 2010, 2015 and 2020 were calculated. By analyzing the results of 2008, 2010, 2015, 2020 optimization calculations, it can be concluded that the total water supply can meet the total water demand in 2008, 2010 and 2015, and the total water supply cannot meet the total water demand in 2020; a shortage of tap water could occur between 2015 and 2020, and the shortage of tap water increases yearly. If industrial reuse water all used up and 8853 m^3^/d of virescence recycled water would not be used, there would be a shortage of 2638 m^3^/d in tap water in 2015 and a total shortage of water supply is 17536 m^3^/d in 2020. If 7885 m^3^/d of virescence recycled water would not be used, there is a serious shortage of 25240 m^3^/d in tap water in 2020.

The solutions of multi-objective optimization model were obtained with acceptable tradeoff among the major variables. Due to the limitation of space, not all the results could be enumerated one by one. So, the result of Daliuta (k = 1) is listed in Table 6 as a representative. In general, the total water supply in 2008, 2010, 2015 and 2020 can meet the water demand of production, domestic, industry and virescence. In 2008, industrial reuse water can meet the requirements of production water demand in the case of non-use of tap water; all domestic water comes from tap water; if the mine does not use tap water, the remaining industrial reuse water can also fully meet the requirements of industrial water demand; all irrigation water comes from the use of the mine’s own virescence recycled water. In 2010, industrial reuse water can meet the requirements of production water demand, in the case of a small amount use of tap water; all domestic water comes from tap water; even if all the remaining industrial reuse water is used for industry, 5375 m^3^/d tap water is still needed to meet the industrial water demand; all irrigation water comes from the use of the mine’s own virescence recycled water. In 2015, industrial reuse water can meet the requirements of production water demand, in the case of a small amount use of tap water; all domestic water comes from tap water; even if all the remaining industrial reuse water is used for industry, 5348 m^3^/d tap water is still needed to meet the industrial water demand; all irrigation water comes from the use of the mine’s own virescence recycled water. In 2020, industrial reuse water can fully meet the requirements of production water demand; all domestic water comes from tap water; even if all the remaining industrial reuse water is used for industry, 6413 m^3^/d tap water is still needed to meet the industrial water demand; all irrigation water comes from the use of the mine’s own virescence recycled water.

**Table 6:** The results of optimal allocation schemes of water resources in Daliuta (unit: m^3^/d).

Planned level years	User	Water supply	Water demand				Surplus or Shortage
	Sources		Production	Domestic	Industry	Virescence	
2008	T	10000	0	2378	0	0	7622
	I	4920	2972	/	892	0	1056
	V	6000	/	/	/	1209	4791
2010	T	10000	12	2644	5375	0	1969
	I	4920	3295	/	1618	0	7
	V	6000	/	/	/	1430	4570
2015	T	10000	0	2626	5348	0	2026
	I	4920	3283	/	1637	0	0
	V	6000	/	/	/	1430	4570
2020	T	10000	0	3282	6413	0	305
	I	4920	4102	/	818	0	0
	V	6000	/	/	/	1764	4236

Note: “/”- Do not participate in this type of water supply, T = Tap water, I = Industrial reused water, V = virescence recycled water.

## Discussion and Conclusions

The work analyzed water resources situation in the study region, including the distribution of water resources, did regional groundwater balance calculation, used GM (1, 1) to predict water demands in the following 15 years, and build a multi-objective programming model for optimal water resources allocation management.

In general, national wastewater discharge amounts, coal mine discharge amount, production of coal, utilization amount of coal mine waste, and national rate of coal mine water utilization have linearly increased. The total volume of water resources is 130.64 million m^3^ in the study area, with the amounts of surface water, groundwater, and mine water of 119.7, 80.8, 12.43 million m^3^, respectively. By analysis of groundwater budget, water storage volume decreased by 1.49 million m^3^ in the balance zone during the balance period.

The tap water and industrial recycled water demands in the following 15 years are forecasted by a same dimension gray recurrence dynamic model GM (1, 1) (Fig. 2), where the supply amounts of tap water and industrial water are used as original data. Demands of all kinds of water resources will increase year by year, the total amount of water demand increases from 4.86 × 10^4^ m^3^/d in the first year of balance duration (2005) to 11.68 × 10^4^ m^3^/d in 2020, in which tap water demand increases by 4.24 × 10^4^ m^3^/d, and industrial recycled water demand increases by 1.86 × 10^4^ m^3^/d. All water demands including domestic, agriculture, industry and mine water consumption, are increasing year by year by a dynamic gray model GM (1, 1). The forecasted mine water drainage is increasing yearly on every mine in the Shendong area by using the hydrogeological analogy method, from 5.13 × 10^4^ m^3^/d in the third year to 6.92 × 10^4^ m^3^/d in the fifteenth year.

The optimal technical schemes among different users are acquired from all nine technical schemes through the application of a multi-objective programming model. The optimal technical schemes among different users are acquired from all nine technical schemes through the application of a multi-objective programming model. By analyzing the results of 2008, 2010, 2015, 2020 optimization calculations, it can be concluded that the total water supply can meet the total water demand in 2008, 2010 and 2015, and the total water supply can’t meet the total water demand in 2020; a shortage of tap water could occur in 2015 and 2020, and the shortage of tap water is increasing year by year. In general, the total water supply in 2008, 2010, 2015 and 2020 can meet the water demand of production, domestic, industry, and virescence.
